# Improving Colon Cancer Prevention in Poland. A Long Way Off

**DOI:** 10.1007/s13187-020-01860-9

**Published:** 2020-09-04

**Authors:** Karolina Obiała, Justyna Obiała, Krzysztof Jeziorski, Jakub Owoc, Małgorzata Mańczak, Robert Olszewski

**Affiliations:** 1 Department of Gerontology, Public Health and Didactics, National Institute of Geriatrics, Rheumatology and Rehabilitation, 1 Spartanska Street, 02-637 Warsaw, Poland; 2 Maria Sklodowska-Curie National Research Institute of Oncology, Warsaw, Poland; 3 Department of Ultrasound, Institute of Fundamental Technological Research, Polish Academy of Sciences, Warsaw, Poland

**Keywords:** Colon cancer, Prevention, Primary healthcare, Education, Communication

## Abstract

The aim of this study was to analyse knowledge on colon cancer prevention among patients of primary care and identify their sources of information. The questionnaire study was conducted among patients of 36 primary healthcare clinics in Poland between September 2018 and February 2019. Patients were interviewed separately by trained researchers. Over 39% of the primary health patients declared that their knowledge about colon cancer prevention is unsatisfactory. Information about colon cancer prevention varied according to sex, age and BMI. Men declared lower level of knowledge than women: 46% of men thought it was unsatisfactory compared with 36% of women (*p* = 0.003). Preventive recommendations were more often provided to patients over 60 years old (*p* < 0.01). Overweight and obese patients were more likely to receive recommendations on diet (*p* < 0.001) and physical activity (*p* < 0.001) than patients with normal weight. The most common source of information on colon cancer prevention was Internet (68%) and medical doctors (60%). There is a need for developing colon cancer prevention policy. Crucial aspect includes educational programs aimed at improving patient’s knowledge and involving medical staff. The policymakers should pay greater attention to cancer prevention policies and medical staff involved in prevention to quality of communication to make sure patients thoroughly understand information they are provided.

## Introduction

Colon cancer accounts for the majority of digestive system cancers. Although its mortality has been decreasing in the twenty-first century, the incidence rate is on the rise, and it is already one of the most common types of cancer among men and women [1, 2]. Prevention is the key in reducing risk of colon cancer as the leading risk factors for sporadic colorectal cancer such as obesity, high total caloric intake, high red meat consumption, high saturated fat intake, excess alcohol consumption, smoking and sedentary lifestyle are all modifiable [3].

The aim of this study was to analyse knowledge on colon cancer prevention among primary care patients and identify their sources of information.

## Methods

The study was conducted in 36 primary healthcare clinics from central Poland in which 509 patients were asked about their opinion on receiving recommendations on proper nutrition, physical activity and medication care. The study was a part of a larger project investigating the role of primary healthcare clinics in education and prevention of diet-related diseases. A self-administered questionnaire for patients was used by trained researchers. The questionnaire consisted of socio-demographic data, lifestyle information including body mass index (BMI), history of colonoscopy within last year and patients’ sources of information on colon cancer prevention. The data on socio-demographics (sex, age, education, place of residence) and lifestyle (smoking status, attitude to health) were categorized into groups: age (≤ 60 and > 60 years of age) and place of residence (village or small town, medium-size town, city).

Information on weight and height of patients was used to calculate BMI. We categorized BMI based on the WHO classification: underweight (BMI< 18.5), normal weight (18.5–24.9), overweight (25.0–29.9) and obese (≥ 30) [4].

The statistical analysis was carried out with Statistica 13.0. The normal distribution of continuous variables was verified using the Shapiro-Wilk test. Continuous data were presented as median and interquartile range (IQR), categorical variables as number and percentage.

The chi-square test was used to assess differences in sociodemographic, lifestyle and health characteristics in various groups of patients. A *p* value of *p* < 0.05 was considered statistically significant.

Ethical approval was obtained from the Ethical Committee of the National Institute of Geriatrics, Rheumatology and Rehabilitation.

## Results

Most of the participants were female (70%, *n* = 354), with secondary (43%, *n* = 220) and higher (44%, *n* = 224) level of education, living in cities (34%, *n* = 174) and villages or small towns (39%, *n* = 199). The median age of patients was 44 (IQR 29–55), and the median BMI was 24.7 (IQR 21.8–28.0) classified as normal weight (Table 1).

**Table 1:** Characteristics of patients

Patients’ characteristics		
Age (years), Me(IQR)	44	(29–55)
Gender (female), *n* (%)	354	(69.5%)
Education, *n* (%)		
Elementary/vocational	65	(12.8%)
Secondary	220	(43.2%)
Higher	224	(44.0%)
Place of residence, *n* (%)		
Village or small town	199	(39.1%)
Medium-size town	136	(26.7%)
City	174	(34.2%)
BMI, Me (IQR)	24.7	(21.8–28.0)
Underweight	11	(2.2%)
Normal weight	263	(51.8%)
Overweight	155	(30.5%)
Obesity	79	(15.6%)
Smokers, *n* (%)	110	(21.6%)

**Fig. 1:**
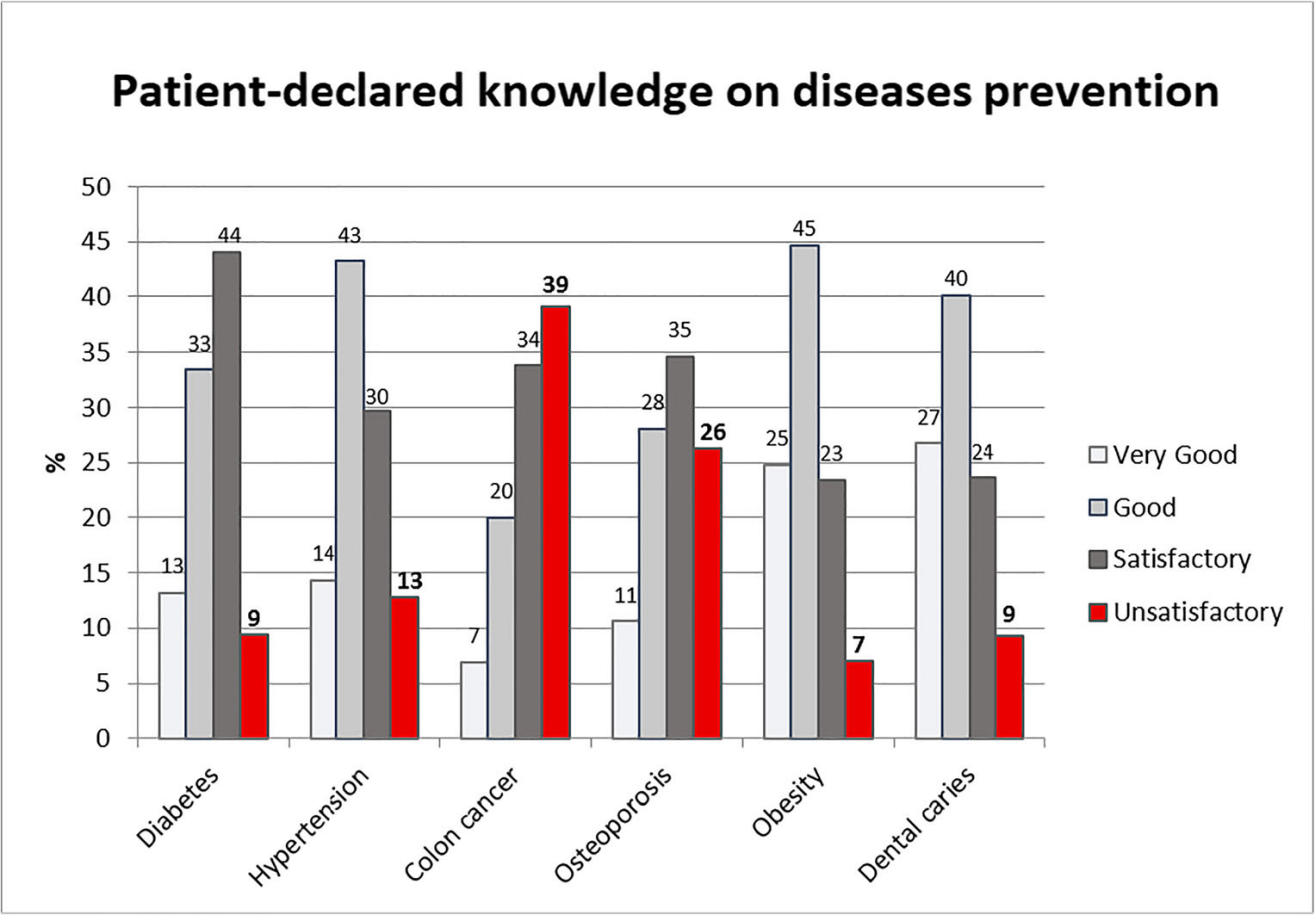
Patient-declared knowledge on disease prevention

The distribution of replies regarding patients’ knowledge about prevention of various diseases varies significantly (*p* < 0.001). Knowledge on colon cancer is very good, good or satisfactory among just 7%, 20% and 34% patients, respectively, which are the lowest ratings for all the presented diseases. The highest level of unsatisfactory knowledge (39%) also refers to colon cancer (Fig. 1).

Information about colon cancer prevention varied according to sex, age and BMI. Men declared lower level of knowledge than women: 46% of men thought it was unsatisfactory compared with 36% of women. As very good, good and satisfactory knowledge declared 9%, 23% and 32% women, respectively, while as very good, good and satisfactory knowledge declared 3%, 13% and 37% men, respectively (*p* = 0.003). Preventive recommendations were more frequently provided to patients over 60 years old. They received recommendations about diagnostic procedures (*p* < 0.001), diet (p < 0.001) and physical activity (*p* = 0.004) more often than patients below 60 years old.

Overweight and obese patients were more likely to receive recommendations on diet (*p* < 0.001) and physical activity (*p* < 0.001) than patients with normal weight; however, they were less likely to comply to these recommendations than patients with normal weight (*p* < 0.001).

Patients with higher education were more likely to be physically active than patients with lower level of education. Daily physical activity was declared by 41%, 35% and 20% respondents with higher, secondary or elementary education, respectively, while “at most once weekly” physical activity was declared by 59%, 65% and 80% patients with higher, secondary and elementary education, respectively (*p* = 0.008). Nevertheless, merely a slight majority of patients with higher education (53%) knew about a preventive role of physical activity in colon cancer (*p* < 0.001).

The most common source of information on colon cancer prevention were Internet (68%) and medical doctors (60%). This information varied according to sex and education. Women (73%) more frequently than men (56%) obtained such information from the Internet (*p* < 0.001). The statistical significance was also observed in case of books, handbooks and press. Women (54%) obtained information on colon cancer prevention from these sources more often than men (28%) (*p* < 0.001) (Table 2).

**Table 2:** Association of patients’ characteristics with their sources of information on colon cancer prevention

Sources of information on colon cancer prevention	Female		Male		*p* value
	*N*	%	*N*	%	
Books, handbooks and press	190	54	44	28	< 0.001
Television, radio	124	35	49	32	0.454
Internet	257	73	87	56	< 0.001
Leaflets and brochures	107	30	39	25	0.245
Medical doctors	200	57	103	66	0.035
Nurses	54	15	17	11	0.199
Dieticians	22	6	5	3	0.242
Events promoting health	15	4	4	3	0.514
Family, friends	136	38	63	41	0.636

## Discussion

The key finding of this study is that patients declared their level of knowledge to be unsatisfactory. Although the survey included both non-modifiable (demographics: age, sex, marital status, etc.) and modifiable factors (screening attitudes, perception of risk for developing colon cancer), the crucial findings refer to the latter. This is in line with the findings of Bidouei et al. [5] who found that over 90% of patients referred to the Razavi Hospital of Mashhad in Iran had no knowledge of colon cancer and screening tests. A study from Hungary [6], in which 81.2% of the responders were not well informed about risk factors, corresponds with these observations. Both of the studies emphasize the issue of education, concluding that the higher the education level, the higher the level of awareness. Our results confirmed this as the patients with higher education were more likely to be physically active than patients with lower level of education. The English, cross-sectional study by Lynes et al. indicated that awareness of risk fctors for colorectal cancer should be improved among younger people [7]. Nevertheless, some Western studies showed that the patients’ knowledge of colon cancer is sufficient. In a study by Moreno et al. [8], the majority of responders (55%) were aware of the general information about colorectal cancer, and 99% believed that colorectal cancer screening was a good idea. Harewood et al. studied not only patients’ awareness of colon cancer prevention in Ireland but also costs of colonoscopy. Majority of patients (91%) were prepared to pay 300 euros for a next-day colonoscopy if recommended by their doctor compared with 7% who preferred to wait 6 months for a free colonoscopy. The age and ethnic differences may play role in patients’ knowledge about colon cancer [9]. The study of Sanchez et al. demonstrated a lower level of knowledge among Hispanic border population in New Mexico compared with non-Hispanic Whites living in the same region [10].

The most common source of information on colon cancer prevention in Poland were Internet (68% of the responders) and medical doctors (60% of responders). While Hungarians used Internet equally often (74.2% of responders), only 36.2% of them pointed to general practitioners or specialists as their source of knowledge [6]. Different results also applied to Italy where sources of knowledge were indicated in the following order: (1) friends, (2) television, (3) newspapers, (4) general practitioners and (5) specialist [11] . Whereas we found sources of information to vary according to sex and education level, in Italy, they were associated with age. Older participants collected information from the general practitioners, specialists, newspapers and brochures more often than younger ones who preferred online sources.

The knowledge of patients about colorectal cancer was strongly associated with patients’ communication with healthcare providers. Patients who rated their patient-provider communication as good were more likely to have completed colorectal screening tests than those reporting poor communication [12]. Moreover, inadequate knowledge among medical staff may be one of barriers affecting colorectal cancer screening rates [13]. One of the Romanian study found that health professionals did not at all contribute to providing patients with information about screening methods in colorectal cancer [14]. Different types of media were the leading sources of information in that population followed by the interpersonal contacts (family and friends).

Our results indicated that majority of primary care patients in central Poland were unaware of colon cancer prevention and their knowledge on proper diet and physical activity was inadequate. Interestingly, this is also relevant for Polish medical students of the Lublin Medical University as majority of them revealed certain deficits in knowledge on colorectal cancer prevention such as selection criteria, access to the care program or screening tests [15].

The development of colon cancer prevention policy is strongly recommended. As the most common sources of information were physicians and Internet, designing educational programs involving medical staff and media may be crucial in improving patient’s knowledge of colon cancer prevention. The results suggest that the policymakers should pay greater attention to cancer prevention policies. On the other hand, medical staff involved in prevention should pay greater attention to quality of communication to make sure patients thoroughly understand the information they are provided.
